# Editorial: Advances in adult neurogenesis

**DOI:** 10.3389/fnins.2023.1307844

**Published:** 2024-01-05

**Authors:** Seiji Hitoshi, Chitra D. Mandyam, Ashok K. Shetty

**Affiliations:** ^1^Department of Integrative Physiology, Shiga University of Medical Science, Otsu, Japan; ^2^VA San Diego Healthcare System, Veterans Health Administration, United States Department of Veterans Affairs, San Diego, CA, United States; ^3^Department of Cell Biology and Genetics, Institute for Regenerative Medicine, Texas A&M University School of Medicine, College Station, TX, United States

**Keywords:** adult neurogenesis, neural stem cells, dentate gyrus, olfactory bulb, V-SVZ

Based on tremendous and elaborate work by Ramón y Cajal and other neuroanatomists around the beginning of 20th Century, it was generally believed that “once the development was ended, the fonts of growth and regeneration…dried up irrevocably (Cajal, 1991).” Pioneering studies by Joseph Altman in the 1960s, which unequivocally showed the generation of new neurons in the hippocampal dentate gyrus of adult mammals by [3H]-thymidine autoradiography (Altman, 1969), were, therefore, underappreciated, and adult neurogenesis remained a controversial field for more than a decade. It was not until the 1980s that the idea of adult neurogenesis was widely accepted following the development of several cell lineage markers and a culturing method of multipotent neural precursor cells from the adult brain (Lendahl et al., 1990; Reynolds and Weiss, 1992). Since then, this field has attracted significant attention, producing a multitude of papers spanning from basic mechanisms underlying the maintenance of the neurogenic niche to the functional significance of neurogenesis in the adult brain in healthy conditions or in a variety of diseases states.

We collected five review papers in this Research Topic of *Frontiers in Neuroscience*. Lampada and Taylor summarize our current knowledge regarding crucial roles of Notch signaling in maintaining neural precursor cell population in the ventricular-subventricular zone (V-SVZ) in the adult brain where quiescent, self-renewing neural stem cells reside. A comprehensive review by Singh et al., outlines transcription factors that play critical roles in preserving neural progenitor activity and promoting subsequent neurogenesis. A review by Inada et al., sheds light on a currently overlooked brain region, the subcommissural organ, and describes its potential in maintaining a neurogenic niche and stimulating neurogenesis in the adult brain. Since it was recognized that thousands of new neurons are born each day, although many of which die within several weeks, and a substantial number of them are incorporated into the granular cell layer of the adult hippocampus in rodents as well as in humans (Spalding et al., 2013), the possible function of the adult dentate neurogenesis in the memory formation and eradication has been extensively investigated. An excellent review by Fölsz et al., introduces preceding important studies and discusses the current situation of this research field. In contrast to the physiological function, a review by Kasahara et al., discusses aberrant neurogenesis in a pathological condition, such as epilepsy. As a whole, the review articles covered in this Research Topic update the state of the adult neurogenesis field, which we believe will help instigate future function questions and studies from scientist's novice to the field.

Seven original research papers also contribute to this Research Topic. Fang et al., provide novel data, demonstrating that one of the tissue inhibitors of metalloproteinases (TIMPs), TIMP3, promotes the maintenance of neural stem cells (NSCs). When newborn neurons are integrated into the existing neural network in the olfactory bulb, Sawada et al., demonstrate that PlexinD1 signaling plays a significant role. Olfactory bulb neurogenesis affects animal behavior when it is disturbed by chronic mild stress, which is shown by Athanassi et al.. Three groups scrutinized the dentate gyrus neurogenesis: Ohyama et al., propose phosphorylated Smad3-positive cells as a distinct subpopulation in the dentate gyrus; Kasakura et al., examined the effects of NT-3 overexpression on the differentiation of dentate gyrus neural precursor cells; and Amelchenko et al., provide evidence showing the correlation between age-related decline in cognitive flexibility and reduced hippocampal neurogenesis. Moreover, an article by Bazarek et al., demonstrates that overexpression of Neurogenin2 could modulate cortical oligodendrocyte progenitor cells to transdifferentiate into neurons, which could lead to a new strategy for functional recovery of the brain following injury or disease in the future.

In sum, this Research Topic aims to clarify what is vigorously explored in the Adult Neurogenesis field, and it would be our pleasure if it draws the attention of young neuroscientists and encourages them to explore this field and contribute studies supporting to the significance of neurogenesis in the adult brain.

## Author contributions

SH: Writing – original draft. CM: Writing – review & editing. AS: Writing – review & editing.
